# Examining DNA fingerprinting as an epidemiology tool in the tuberculosis program in the Northwest Territories, Canada

**DOI:** 10.3402/ijch.v72i0.20067

**Published:** 2013-05-08

**Authors:** Cheryl Case, Kami Kandola, Linda Chui, Vincent Li, Nancy Nix, Rhonda Johnson

**Affiliations:** 1 Health Sciences, University of Alaska, Anchorage, AK, USA; 2 Stanton Territorial Health Authority, Yellowknife, NT, Canada; 3 Department of Health and Social Services, Government of Northwest Territories, Yellowknife, NT, Canada; 4 Department of Laboratory Medicine and Pathology, University of Alberta Hospital, Edmonton, AB, Canada

**Keywords:** tuberculosis transmission, RFLP, social network analysis, aboriginal, DNA fingerprinting, contact tracing

## Abstract

**Background:**

Tuberculosis (TB) is an important public health problem in the Northwest Territories (NWT), particularly among Canadian Aboriginal people.

**Objective:**

To analyse the transmission patterns of tuberculosis among the population living in the NWT, a territorial jurisdiction located within Northern Canada.

**Methods:**

This population-based retrospective study examined the DNA fingerprints of all laboratory confirmed cases of TB in the NWT, Canada, between 1990 and 2009. An isolate of each lab-confirmed case had genotyping done using IS*6110* Restriction Fragment Length Polymorphism. DNA patterns were assigned to each DNA fingerprint, and indistinguishable fingerprints patterns were assigned a cluster. Social network analysis (SNA) was used to examine direct linkages among cases determined through conventional contact tracing (CCT), their DNA fingerprint and home community.

**Results:**

Of the 225 lab-confirmed cases identified, the study was limited to 195 subjects due to DNA fingerprinting data availability. The mean age of the cases was 43.8 years (±22.6) and 120 (61.5%) males. The Dene (First Nations) encompassed 120 of the cases (87.7%), 8 cases (4.1%) were Inuit, 2 cases (1.0%) were Metis, 7 cases (3.6%) were Immigrants and 1 case had unknown ethnicity. One hundred and eighty six (95.4%) subjects were clustered, resulting in 8 clusters. Trend analysis showed significant relationships between with risk factors for unemployment (*p*=0.020), geographic location (*p*≤0.001) and homelessness (*p*≤0.001). Other significant risk factors included excessive alcohol consumption, prior infection with *Mycobacterium tuberculosis* and prior contact with a case of TB.

**Conclusions:**

This study demonstrates how DNA fingerprinting and SNA can be additional epidemiological tools, along with CCT method, to determine transmission patterns of TB.

Tuberculosis (TB) is an important public health problem in the Northwest Territories (NWT), particularly among Canadian Aboriginal people. TB was first reported in the NWT by the early missionaries in the later years of the 19th century (1). TB was epidemic in the early 1940s in the NWT with a reported 42 deaths per 10,000 population (1). TB continues to be an endemic disease among the Aboriginal population (Dene, Inuit and Metis) who comprise roughly half of the NWT population.

Despite effective antibiotic treatment, standardised clinical management programs and rigorous contact tracing, the rate of TB in the NWT averages 20 cases per 100,000 population (2), 4 times the national rate (3). Outbreaks have been reported among populations living in remote communities throughout the NWT. The TB rates among the Aboriginal population are twice the overall NWT rate (2). Continued transmission of this disease can be attributed to late identification of a respiratory case of TB resulting in subsequent progression of the disease to an infectious advanced stage allowing high amounts of *M. tuberculosis* in the respiratory tract to be expelled into the air. Conventional contact tracing (CCT) remains an important method to stop the chain of transmission of TB in the NWT.

DNA fingerprinting is a tool that can be used to evaluate gaps in the CCT method and determine clonal relatedness of *M. tuberculosis* isolates (4–6). The case and their infected contacts have the same indistinguishable DNA fingerprint. Contact tracing investigations are significantly enhanced if TB cases share an indistinguishable DNA fingerprint typing in addition to the traditional epidemiological links as determined through CCT.

Another useful approach is social networking analysis (SNA), which is a mathematical tool that includes visualisation of people and places and the connections between them (7–9). Due to the lengthy latency period of TB and the mode of transmission through the air, CCT may not capture all of the contacts. SNA has been used to determine socialising patterns by directing focus on locations and activities contributing to potential transmission (10).

The objective of this study was to better understand the transmission patterns of tuberculosis among the Northern Canadian population living in the NWT.

The aims of this study were to determine: (a) whether unknown transmission among the studied cases not previously identified through CCT can be identified by examination of DNA fingerprinting patterns; and (b) whether specific TB risk factors related to demographics, social and behavioural risk factors, and clinical aspects are associated with DNA fingerprinting patterns.

## Materials and methods

We conducted a 20-year retrospective population-based study examining DNA fingerprinting patterns of isolates from reported NWT TB cases between January 1990 and December 2009 matched to the epidemiological and demographic data. DNA fingerprinting analysis of each *M. tuberculosis* isolate corresponded to a single TB case reported during the study period.

### Epidemiological data

Demographic and epidemiologic data were obtained from medical records of all patients diagnosed with TB at the Office of the Chief Public Health Officer (OCPHO). All data were collected by staff at the OCPHO and stored in hard copy and electronic copy in the integrated Public Health Information system (iPHIS), a web-based data management application.

Demographic, social and behavioural risk factors, and clinical aspects included: age, gender, ethnicity, employment status, amount of alcohol consumption, illicit drug use, smoking, homeless status, HIV and past TB exposure history including prior contact with an active TB case and previous latent tuberculosis infection (LTBI).

### DNA fingerprint analysis

Molecular typing method for genotyping of the NWT *M. tuberculosis* isolates has been a routine procedure at the Provincial Laboratory for Public Health (ProvLab), Alberta Health Services even prior to the onset of this study. The ProvLab uses an international standardised protocol for IS*6110* restriction fragment length polymorphism [IS*6110-*RFLP] (11). Images of the IS*6110*-RFLP patterns were digitized and stored in databases managed using the BioNumerics software (version 5.1; Applied Maths, USA). RFLP fingerprint pattern numbers were assigned to each isolate, and cluster analysis was performed with BioNumerics. Dendrograms were made using BioNumerics using the unweighted pair group method with arithmetic mean, a Dice similarity coefficient, an additional 1.0% similarity coefficient and 1.5% optimisation.

### Definition of clustering

A cluster of *M. tuberculosis* isolates included isolates with characteristics of the same number of copies (greater than 5) with IS*6110* fragments of identical molecular weight and greater than 85% band agreement within the timeframe of 1990–2009.

### Statistical analysis

Data were analysed using Statistical Package for Social Services software version 17.0 (SPSS Inc., Chicago IL). Univariate analysis of the potential TB risk factors of each case of TB was examined by grouping the genotype from their matched isolate into DNA fingerprint clusters or not clustered (unique). The association of each risk variable (demographic, social and behavioural risks and clinical aspects) was compared to the outcome variable of DNA fingerprint cluster groupings. Bivariate analysis was used to test association using Chi-squared test or Fisher's exact test. P values <0.05 were considered as statistically significant. Strength in the statistical power was increased by grouping the DNA clusters as: the 2 dominant DNA clusters and grouping the remaining cases belonging to other clusters and unique DNA fingerprints as the outcome variable.

### Social network analysis

SNA permitted the visualisation of patterns or connections between cases and communities focused on the 2 dominant DNA fingerprint clusters. SNA was used as a tool to examine TB transmission within a population due to person-to-person, person-to-place mapping and showing recent transmission. Recent transmission was defined as having the 2 cases reported within 2 years. Examination of known exposure, based on CCT records of each case, was examined through the iPHIS database. The system allowed each case to be cross-referenced with reported contact to other cases. PAJEK (12), a SNA application, was used for visualising network analysis to measure the connections between cases and communities. Both methods, SNA and CCT can detect evidence of transmission but depending on the socialisation patterns of the case(s) being studied, one or both methods may provide more conclusive findings of transmission patterns (7).

The research proposal was reviewed and approved by the University of Alaska Anchorage Institutional Review Board and the Aurora Research institute (Research Licence # 1280, NWT).

## Results

Between 1 January 1990 and 31 December 2009, there were 225 laboratory-confirmed cases reported in the NWT. However, the study was limited to 195 subjects because the DNA fingerprint data were not available for 30 of the isolates at the laboratory. Clustering analysis was performed on isolates with IS*6110* RFLP data, incorporating 95% (186/195) of the cases in this study and grouped into clusters labelled: NWT1–NWT8 (Table I).

**Table I:** DNA cluster frequencies

Assigned cluster number	Number grouped into cluster	Percent
NWT1	79	40.5
NWT2	78	40.0
NWT3	10	5.1
NWT4	8	4.1
NWT5	5	2.6
NWT6	2	1.0
NWT7	2	1.0
NWT8	2	1.0
Unique DNA fingerprints	9	4.7
Total	195	100

**Fig. 1:**
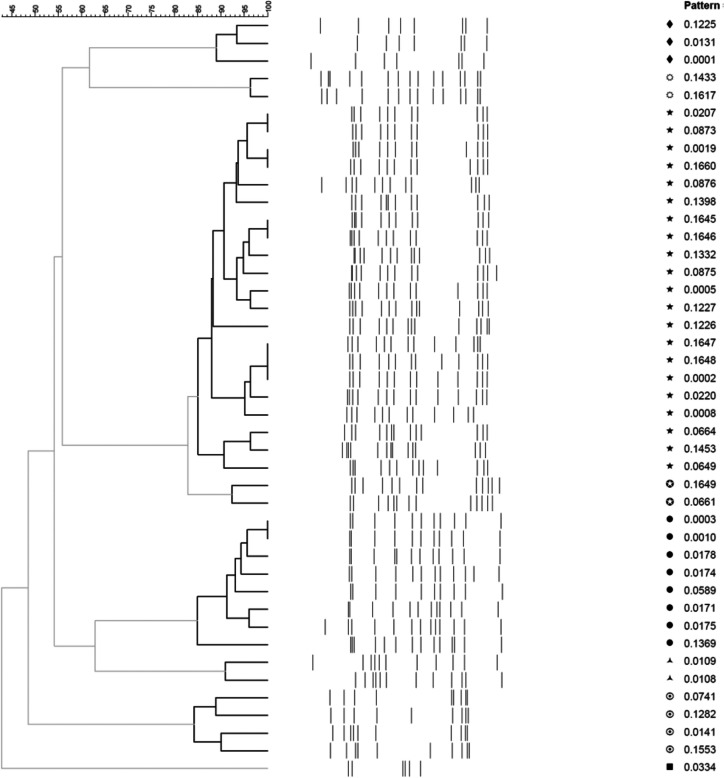
Dendrogram of clustering analysis of the IS*6110* RFLP patterns of the strains in this study. Dendrogram nodes and associated sub-branches that fit the cluster definition (≥85% pattern similarity) are highlighted in bold. The NWT clusters are represented as follows: 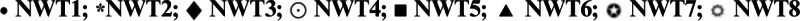.

Figure 1 demonstrates a dendrogram of clustering analysis of the IS*6110* RFLP patterns of the strains in this study.

The 2 dominant DNA fingerprint clusters were NWT1 and NWT2, and they included 40.5% (79/195) and 40.0% (78/195) of the isolates, respectively (Table I). The clusters NWT3 to NWT8 and an additional 9 unique DNA fingerprints (did not cluster) were amalgamated into a grouping called “others”. Detailed case characteristics are shown in Table II.

**Table II:** Description of case's characteristics from DNA clusters and bivariate analysis

		DNA cluster grouping					
		
Case characteristics		NWT1 *n*=79	NWT2 *n*=78	All others *n*=38	NWT1 versus NWT2 (*p*-value)	NWT1 versus all others (*p*-value)	NWT2 versus all others (*p*-value)
Mean Age (SD)		42 (24)	44 (23)	48 (20)	0.047	0.137	0.814
Gender (%)	Male	43 (54)	50 (64)	27 (71.0)	0.126	0.064	0.299
	Female	36 (46)	28 (36)	11 (29.0)			
Ethnicity (%)	Dene	75 (94.9)	71 (91.0)	26 (68.4)	0.548*	<0.001*	<.001
	Inuit	1 (0.7)	1 (1.3)	6 (15.8)			
	Metis	0 (0)	2 (2.6)	0 (0)			
	Non-Aboriginal	3 (3.7)	3 (3.8)	0 (0)			
	Immigrant	0 (0)	1 (1.3)	6 (15.8)			
	Missing	1 (0.7)	0 (0)	0 (0)			
Employment (%)	Yes	45 (57.0)	35 (44.9)	23 (60.5)	0.054	0.681	0.020
	No	12 (15.2)	27 (34.6)	8 (21.0)			
	Child/student	12 (15.2)	10 (12.8)	4 (10.5)			
	Missing	10 (12.6)	6 (7.7)	3 (8.0)			
Community –	A	29 (36.7)	18 (23.1)	2 (5.3)	0.001*	<0.001*	<0.001*
major (%)	B	27 (34.2)	1 (1.3)	0 (0)			
	C	7 (8.9)	24 (30.8)	11 (28.9)			
	Others	16 (20.2)	35 (44.8)	25 (65.8)			
Excessive Alcohol	Yes	35 (44.3)	41 (52.6)	15 (39.5)	0.065	0.485	0.120
	No	40 (50.6)	29 (37.2)	19 (50.0)			
	Missing	4 (7.6)	8 (10.2)	4 (10.5)			
Drug use	Yes	27 (34.2)	24 (30.8)	13 (34.2)	0.487	0.489	0.436
	No	46 (58.2)	44 (56.4)	20 (52.6)			
	Missing	6 (7.6)	10 (12.8)	5 (13.2)			
Homeless	Yes	3 (3.8)	16 (20.5)	4 (10.5)	0.003	0.313	<0.001
	No	70 (88.6)	55 (70.5)	34 (89.5)			
	Missing	6 (7.6)	7 (9.0)	0 (0)			
Smoker	Yes	15 (19.0)	14 (18.0)	2 (5.3)	0.574	0.060	0.057
	No	53 (67.1)	48 (61.5)	27 (71.0)			
	Missing	11 (13.9)	16 (20.5)	9 (23.7)			
HIV	Positive	0 (0)	0 (0)	0 (0)	0.536	0.144	0.130
	Negative	40 (51.6)	40 (51.3)	15 (39.5)			
	Missing	39 (49.4)	38 (48.7)	23 (60.5)			
Latent tuberculosis infection	Yes	40 (50.6)	36 (46.1)	18 (47.4)	0.213	0.299	0.574
	No	38 (48.1)	42 (53.8)	20 (52.6)			
Prior contact	Yes	44 (55.7)	40 (51.3)	7 (18.4)	0.262	<0.001	0.001
	No	35 (44.3)	38 (48.7)	31 (81.6)			
Pulmonary TB	Yes	68 (86.1)	69 (88.5)	31 (81.6)	0.500	0.473	0.337
	No	11 (13.9)	9 (11.5)	7 (18.4)			

* Chi-squared test by Phi, remainder by Fisher exact test.

Due to the presentation of the majority of isolates meeting the clustering definition, the statistical analysis involved comparing the dominant DNA clusters to one another and each of the dominant clusters with the amalgamated grouping titled, “others”. Comparison among the 3 groupings was a method used to determine whether a significant association of DNA fingerprint clustering existed with the risk factors examined in the study. The ethnicity of the cases was primarily Dene among the 2 most dominant clusters, NWT1 and NWT2 representing 75 (94.9%) and 71 (91.0%) of the cases, respectively. Ethnicity frequency among the “other” DNA fingerprints included a higher proportion of cases representing Inuit and Immigrant populations with 6 (15.8%) cases for each. Incorporating all of the examined cases, 87.7% (171/195) were Dene, followed by 4.1% (8/195) Inuit, 3.6% (7/195) Immigrant, 3.1% (6/195) non-aboriginal, 1.0% (2/195) Métis and 0.5% (1/195) of unknown ethnic group. The mean age of the 3 groupings NWT1, NWT2 and “others” was 42, 44 and 48 years, respectively. Gender was evenly distributed among the NWT1 cluster but was predominately male among the NWT2 and “others” groupings.

Unemployment status varied among the 3 grouping, NWT1, NWT2 and “others” with 12 (15.2%), 27 (34.6%) and 8 (21.0%), respectively. Children and students were excluded from this analysis while the employed group included homemakers and retired individuals, assuming these 2 categories that did not seek employment. The TB cases originated from 24 of the 33 communities in the NWT. In the overall analysis of all of the 195 TB cases, the 3 communities representing the highest number of cases were Community A with 21.5% (42/195), Community B with 14.4% (28/195) and Community C with 25.1% (49/195). NWT1 cases were predominately in Communities A and B, representing 36.7 and 34.2%, respectively, while the majority of the cases in the NWT2 cluster and “others” grouping were represented in the remaining communities. Due to the low populations in the isolated communities, anonymity of the community name was required in this study.

Harmful alcohol drinking included those who had reported frequent heavy drinking or a history of alcohol dependency was greater than 39.5% among the 2 dominant DNA clusters and “others” grouping. Homelessness was reported among all 3 groupings with NWT2 having the highest frequency of 16 cases (20.5%).

Clinical aspects of the cases included nearly half of the cases grouped in NWT1, NWT2 and “others” reporting evidence of LTBI, indicating that close to half of the cases may have been reactivations. The majority of the cases were diagnosed with respiratory TB averaging 85%, and the remaining was non-respiratory TB. Approximately half of the cases had recorded HIV testing done, all reported as negative.

Bivariate analysis using Chi-squared and Fisher's exact test were used to examine association among the two dominant DNA fingerprint clusters (NWT1 and NWT2) and the remaining DNA fingerprints as “others”. In Table II, the analysis between NWT1 and NWT2 showed significant association among the risk factors of age (*p*=0.047), community (*p*=0.001) and homelessness (*p*=0.003). NWT1 verses “others” DNA fingerprints had significance for ethnicity (*p*≤0.001), community (*p*≤0.001) and prior contact with a case (*p*≤0.001). NWT2 verses “others” DNA fingerprints showed significance for ethnicity (*p*≤0.001), employment (*p*=0.020), community (*p*≤0.001), homelessness (*p*≤0.000) and prior contact with a case (p<0.001).

**Fig. 2:**
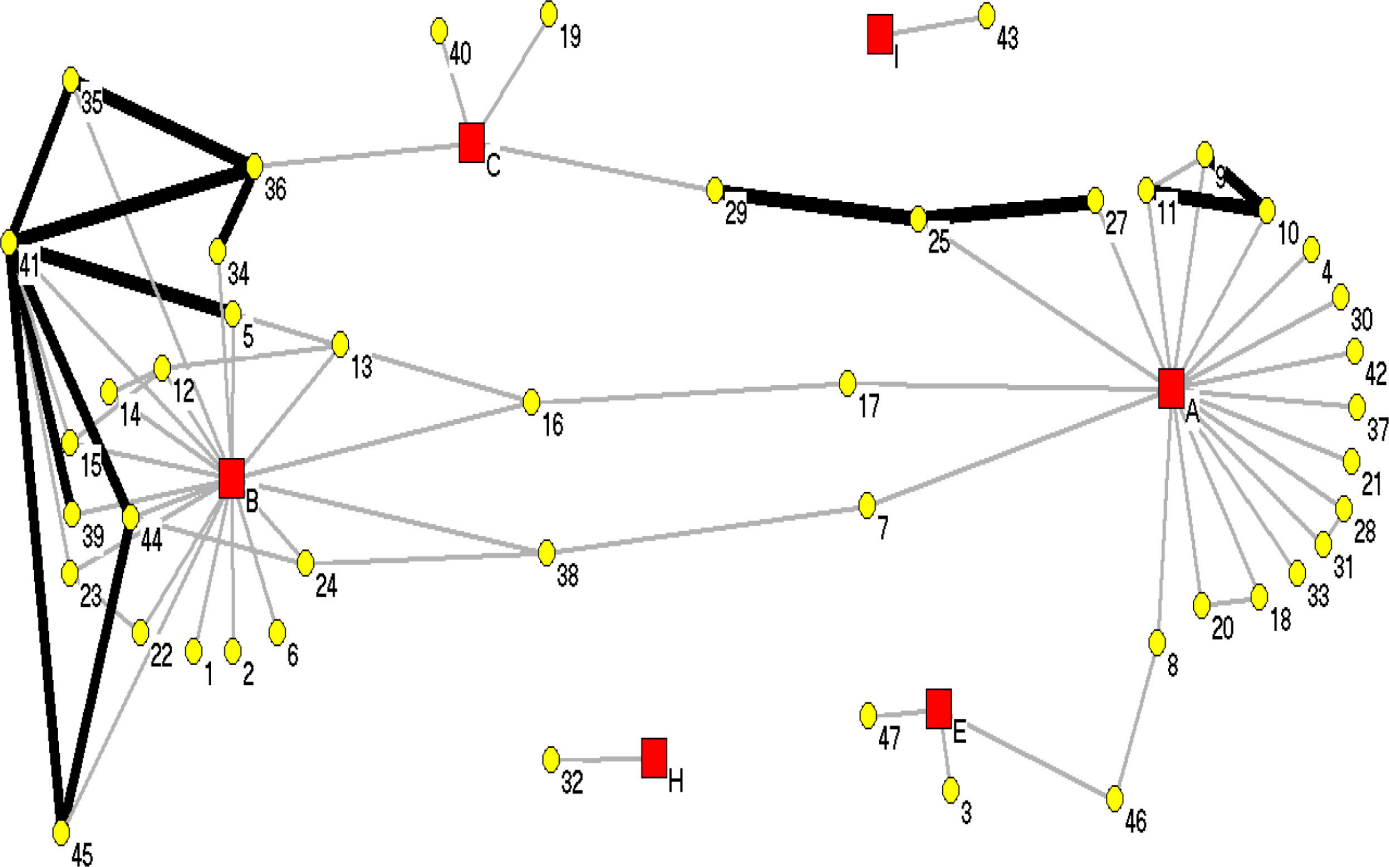
SNA of NWT1 DNA fingerprint cluster. The yellow circles represent the cases and the red squares represent the communities. The heavy black lines represent cases reported within 2 years, while the lighter lines represent exposure between the cases exceeding 2 years.

SNA was done on the cases without reported records of LTBI among the two dominant DNA clusters, NWT1 and NWT2, representing 47 cases in each cluster. These cases were selected primarily to lessen the possibility of the case having previous exposure to cases not included in this study. As well, the cases with exposure to another case within two years were considered recent transmission. In separate SNA examination of the two dominate DNA fingerprint clusters, each case was assigned a unique identification number with the DNA cluster and communities were assigned a unique letter, both referred to as “nodes”. Each case was assigned a colour code for their DNA fingerprint cluster. Figure 2 demonstrates the relationship between cases and their connections with communities for NWT1 DNA cluster.

Figure 2 shows the relationship between the 47 cases reported between 1990 and 2004, with their isolate's DNA fingerprint classified as NWT1. The cases were distributed among six communities (A, B, C, E, H and I), all located around the Great Slave Lake area. Note a few of the cases have multiple heavy black lines, indicating recent transmission among cases. Case “41” was an index case resulting in an outbreak in Community B starting in 1995. Recent contact was reported among cases “5”, “35”, “36”, “39”, “44” and “45”. As well, note the direct link of case “8” between Community A and Community E, cases “7” and “17” to Community B and cases “25” and “27” to Community C. Although many cases were directly linked to one community, the social patterns show spread to other communities.

**Fig. 3:**
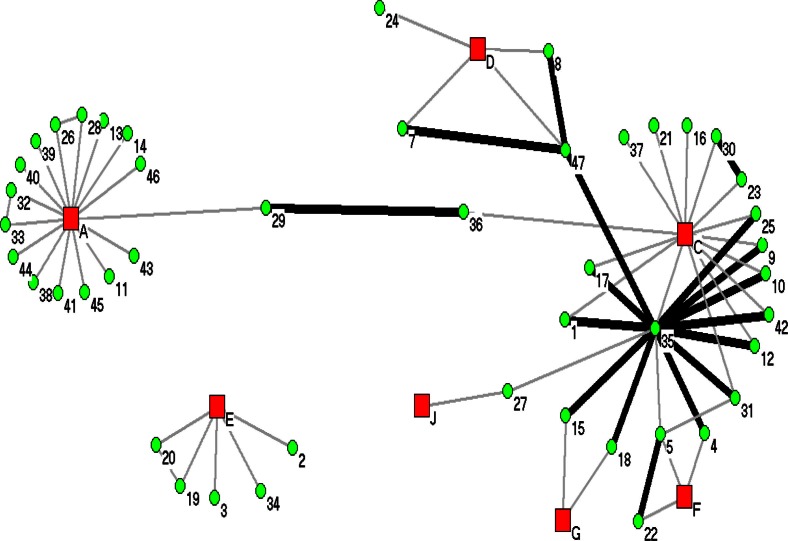
SNA of NWT2 DNA fingerprint cluster. The green circles represent the cases and the red squares represent the communities. The heavy black lines represent cases reported within 2 years, while the lighter lines represent exposure between the cases exceeding 2 years.

Seven communities were associated with the distribution of 47 cases matching to isolates grouped in NWT2, shown in Figure 3. The cases in this figure were reported between 1991 and 2009. NWT2 case numbers do not match with assigned case numbers in NWT1. Case “35” was the index case reported in 2007 from a homeless shelter outbreak in Community C. Twelve cases (“1”, “4”, “9”, “10”, “12”, “15”, “17”, “18” “25”, “31”, “42” and “47” had direct transmission reported within the 2-year criteria as well as having cases linked to 5 communities (Communities A, C, D, F and G).

## Discussion

Early case detection and timely completion of treatment are the most important measures to stop the spread of TB in a community. CCT focuses on the concentric model for determining risk of contracting TB, where household members are usually considered at the highest risk of acquiring the infection (13). The rationale for investigating contacts of a TB patient is that the infection can be spread through airborne droplet nuclei containing *M. tuberculosis* (13). The identification and differentiation of the strains of *M. tuberculosis* by *IS6110*-RFLP has provided a better understanding of the epidemiology of the transmission of TB in the NWT. Although this study did not determine the direction of transmission among cases, it was able to determine associations of indistinguishable DNA fingerprints or clustering with some risk factors such as age, ethnicity, unemployment, excessive alcohol consumption, geographic location, homelessness and previous exposure to TB cases. This study does identify one unknown cluster, NWT8 consisting of 2 cases with indistinguishable DNA fingerprints, not identified through CCT.

The most important outcome of this study is the development of a database of DNA fingerprint patterns on all culture confirmed cases of TB in the NWT for the last 20 years. The DNA fingerprint registry will be invaluable in prospective analysis of outbreaks to assist with linking to known outbreaks and determining new ones. TB is a disease often associated with marginalised populations. In this study, among the 195 cases, over 90% of the cases were of Aboriginal ethnicity, 24.1% unemployed, 46.7% excessive alcohol consumers, 32.8% illicit drug users and 9.7% declared as homeless at some time during the progression of disease and treatment.

In this study, a large proportion of the case's isolate belonged to a cluster, 186/195 (95.4%). Conversely, a 2-year study among cases of TB in the Canadian provinces of British Columbia, Alberta, Saskatchewan and Manitoba only had 32.1% of their cases grouped into clusters (14). The remaining 67.9% were unique DNA fingerprints. DNA fingerprinting homogeneity identified in this study suggests 2 things: first, the population is fairly non-transient in the NWT, meaning the circulating strains of *M. tuberculosis* is limited and endemic, and/or second, there is a high amount of transmissibility among cases in the NWT with endemic strains. The SNA demonstrates that both are feasible explanations.

SNA demonstrated that there is a strong relationship between cases within communities and among other communities. Further study could be done using SNA to demonstrate temporal spacing of the transmission of TB among the study group with further analysis of the genomes of the endemic strains. SNA allows the focus of the investigation to shift from individual case investigations to broader population-based examination of commonalities such as common networks of drug use or places of social congregation.

In conclusion, this study demonstrates how DNA fingerprinting and SNA can be additional epidemiological tools, along with the CCT method, to determine transmission patterns of TB. The 3 tools complement one another and each provides significant additional information to a TB investigation, which could be applied to prospective and retrospective investigations for TB transmission patterns. In this study, TB is most prevalent among marginalised populations in the NWT, and future control efforts need to focus on social networking patterns related to geographic location, alcohol consumption, exposure to a case, unemployment and homelessness.

TB remains a serious problem among the Aboriginal population in the NWT. Over half of the cases had evidence of being infected long before progression to active disease; they had evidence of previous LTBI. A high degree of strain homogeneity and previous infection with *M. tuberculosis* raises the question of whether large-scale testing and treatment of latent infection might be an effective way of dramatically reducing TB rates in some of the isolated communities in the NWT. Another option may be to drill down to the population at highest risk for contracting TB and targeting screening and treatment programs.
